# Vocal Fold Motion Impairment in Neurodegenerative Diseases

**DOI:** 10.3390/jcm13092507

**Published:** 2024-04-24

**Authors:** Rumi Ueha, Cathrine Miura, Naoyuki Matsumoto, Taku Sato, Takao Goto, Kenji Kondo

**Affiliations:** 1 Swallowing Center, The University of Tokyo Hospital, Tokyo 113-8655, Japan; 2 Department of Otolaryngology and Head and Neck Surgery, Faculty of Medicine, The University of Tokyo, Tokyo 113-8655, Japan; miura.cathrine@gmail.com (C.M.); smilenaoyuki24@gmail.com (N.M.); taku.koro.z@gmail.com (T.S.); gottytakao@gmail.com (T.G.); kondok-tky@umin.ac.jp (K.K.)

**Keywords:** vocal fold motion impairment, neurodegenerative diseases, Parkinson’s disease, multiple system atrophy, amyotrophic lateral sclerosis, treatment, surgery

## Abstract

Vocal fold motion impairment (VFMI) is the inappropriate movement of the vocal folds during respiration, leading to vocal fold adduction and/or abduction problems and causing respiratory and vocal impairments. Neurodegenerative diseases (NDDs) are a wide range of disorders characterized by progressive loss of neurons and deposition of altered proteins in the brain and peripheral organs. VFMI may be unrecognized in patients with NDDs. VFMI in NDDs is caused by the following: laryngeal muscle weakness due to muscular atrophy, caused by brainstem and motor neuron degeneration in amyotrophic lateral sclerosis; hyperactivity of laryngeal adductors in Parkinson’s disease; and varying degrees of laryngeal adductor hypertonia and abductor paralysis in multiple system atrophy. Management of VFMI depends on whether there is a presence of glottic insufficiency or insufficient glottic opening with/without severe dysphagia. VFMI treatment options for glottic insufficiency range from surgical interventions, including injection laryngoplasty and medialization thyroplasty, to behavioral therapies; for insufficient glottic opening, various options are available based on the severity and underlying cause of the condition, including continuous positive airway pressure therapy, botulinum toxin injection, tracheostomy, vocal fold surgery, or a combination of interventions. In this review, we outline the mechanisms, clinical features, and management of VFMI in NDDs and provide a guide for physicians who may encounter these clinical features in their patients. NDDs are always progressive; hence, timely evaluation, proper diagnosis, and appropriate management of the patient will greatly affect their vocal, respiratory, and swallowing functions as well as their quality of life.

## 1. Introduction

Neurodegenerative diseases (NDDs) encompass a wide range of disorders characterized by progressive loss of neurons and deposition of altered proteins in the brain and peripheral organs [1,2]. These conditions tend to develop slowly, with symptoms becoming evident in the later stages of life. NDDs can manifest with a range of symptoms, including memory loss, abnormal movements, balance impairments, and swallowing difficulties, as well as potential complications such as voice and breathing disorders, which may emerge either at the onset or throughout disease progression [1,3].

Voice disorders can arise from various neurological conditions that affect the corticospinal pathway, cerebellum, basal ganglia, and upper and/or lower motor neurons [4]. A comprehensive examination of voice disorders in neurological diseases, emphasizing acoustic analysis, underscored the significant role of neurodegenerative diseases in affecting voice quality, especially in movement disorders such as Parkinson’s disease (PD), essential tremor, and spasmodic dysphonia [4,5]. 

Vocal fold motion impairment (VFMI) is also often encountered in NDDs [6,7,8,9]. VFMI is characterized by inappropriate movement of the vocal folds during breathing, leading to difficulties in vocal fold opening or closing. VFMI refers to a condition in which there is a disturbance in the movement of the vocal folds, but the terms vocal fold movement impairment [10], vocal cord (fold) paralysis [11,12], vocal cord (fold) dysfunction [13,14], and paradoxical vocal fold motion [15] are used as synonyms and analogues. 

There are several reviews of voice disorders in NDDs but few reviews of VFMI in NDDs. Many physicians may have limited experience with NDDs and may be largely unaware of VFMI in these patients. Since VFMI related to NDDs is not widely recognized, even among otolaryngologists who assess vocal fold function, physicians may not readily attribute VFMI findings on endoscopic evaluation to NDDs as the underlying pathology. Recognizing VFMI as an early sign of NDDs may prompt otolaryngologists to expedite referrals to neurologists. In addition, if neurologists recognize hoarseness or respiratory difficulties in patients with NDDs, they should anticipate the potential for VFMI and encourage an early laryngeal assessment by otolaryngologists, particularly given the risk of respiratory deterioration.

To inform healthcare professionals about VFMI in patients with NDDs, we have compiled this review article. In this review article, we describe the clinical features and mechanisms of VFMI in NDDs and, additionally, provide insights into therapeutic approaches. Notably, we used the term vocal fold dysfunction to describe a condition in which the vocal folds move differently than expected, vocal fold dysmotility to describe a condition in which the vocal folds have reduced movement, and vocal fold immobility to describe a condition in which the vocal folds hardly move.

## 2. Diagnosis and Etiology of Vocal Fold Motion Impairment

VFMI is diagnosed through the following methods [16,17,18,19,20]:

- Flexible laryngoscopy [16,17];
- Laryngeal electromyography [18,19];
- Ultrasonography [17,20].

Laryngological examination using a flexible laryngoscopy is an important tool for diagnosing VFMI, since it can visualize the vocal fold movement during breathing and phonation [16,17]. VFMI, including vocal fold immobility, vocal fold hypomobility, and vocal fold dysmotility, describes the qualitative findings of vocal fold motion without assuming a specific cause. These terms are preferred for describing physical exam results, especially through flexible laryngoscopy. The terms vocal fold paralysis and vocal fold paresis indicate a neurological cause for the abnormality seen during examination [16]. Laryngeal electromyography is a valuable tool in the evaluation and management of VFMI, providing prognostic information and guiding treatment decisions, as well as monitoring recovery in patients with VFMI. Laryngeal electromyography can differentiate between neuropathic patterns in the laryngeal muscles and other causes of vocal fold immobility, such as arytenoid dislocation [18,19,21]. Laryngeal ultrasonography can be a useful tool for screening and evaluating vocal fold motion impairment, but its accuracy is still debated compared to the standard of flexible laryngoscopy, especially in adult populations [20,22]. 

VFMI can occur in unilateral or bilateral vocal folds, and the etiology includes the following [23,24,25,26,27]:

- Neurological conditions;
- Malignancies;
- Mechanical/structural causes;
- Trauma;
- Iatrogenic injuries;
- Other potential causes.

**Figure 1:**
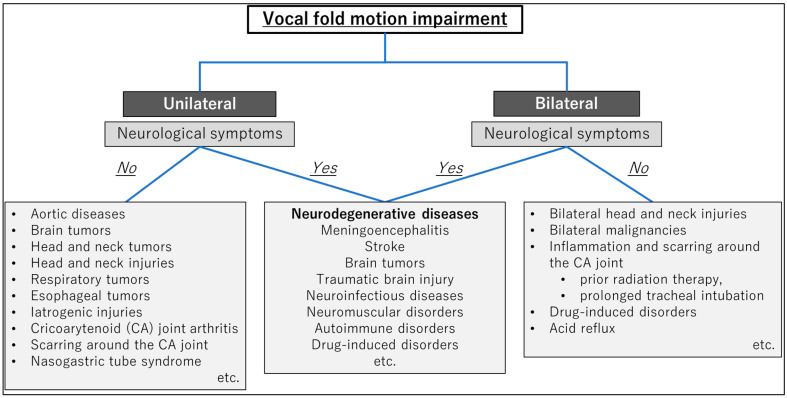
Etiologies of vocal fold motion impairment. CA, cricoarytenoid.

The etiologies of VFMI are summarized in Figure 1. Neurological conditions like stroke, neurodegenerative diseases, and Guillain–Barré syndrome may result in VFMI [23]. Scarring or inflammation around the cricoarytenoid joint can cause mechanical vocal fold immobility [26]. Tumors or malignancies in the neck and laryngeal region can compress or infiltrate the nerves controlling the vocal folds [17]. VFMI can be triggered by factors like exercise, strong odors, acid reflux, and stress, though the underlying causes are not well understood [25].

## 3. Vocal Fold Motion Impairment in Neurodegenerative Diseases

NDDs are classified into the following categories based on their background mechanisms: dementia-type diseases, demyelinating diseases, parkinsonism-type diseases, motor neuron diseases, and prion diseases [2,3,28,29]. Dementia-type diseases include various disorders like Alzheimer’s disease, frontotemporal dementia, chronic traumatic encephalopathy, and dementia with Lewy bodies (DLB). This entails progressive deterioration in distinct brain regions, resulting in neuronal demise in multiple areas [3,30]. Demyelinating diseases are a group of neurological disorders characterized by damage or loss of the myelin sheath, which protects the surrounding nerve fibers in the central and peripheral nervous systems. Some demyelinating diseases, such as multiple sclerosis (MS) and neuromyelitis optica spectrum disorder (NMOSD), can be considered both demyelinating and neurodegenerative because of their impact on axons and neurons [31,32]. Parkinsonism-type diseases are a group of NDDs characterized by motor symptoms, such as bradykinesia, tremor, rigidity, and postural instability. Parkinsonism-type diseases include PD, DLB, progressive supranuclear palsy (PSP), and multiple system atrophy (MSA). Neuronal loss is associated with the accumulation of misfolded proteins, such as α-synuclein in PD and MSA and tau protein in PSP [33,34]. Motor neuron diseases are a subset of neurodegenerative disorders that primarily affect motor neurons and lead to progressive muscle weakness and atrophy. These include amyotrophic lateral sclerosis (ALS), spinal muscular atrophy (SMA), and primary lateral sclerosis (PLS). ALS is associated with the accumulation of the TDP-43 protein, whereas SMA stems from mutations in the survival motor neuron gene [35,36]. Prion diseases are caused by abnormal folding of prion proteins, which leads to extensive brain degeneration, including spongiform changes, neuronal loss, and accumulation of protein aggregates [37]. Prion diseases include Creutzfeldt–Jakob disease (CJD), Gerstmann–Sträussler–Scheinker syndrome, and kuru in humans, as well as scrapie in sheep and goats [38].

Among the NDDs, VFMI is frequently encountered in ALS, PD, MSA, PSP, and MS. Laryngoscopy can assess the movement and function of the vocal folds and identify abnormalities, such as incomplete glottic closure, vocal fold paralysis, or bowing of the vocal folds. Stroboscopy provides a detailed view of vocal fold vibration during phonation, helping to identify subtle abnormalities in vocal fold movement and closure. The following outlines the characteristics of VFMI under each condition.

### 3.1. Amyotrophic Lateral Sclerosis (ALS)

**Figure 2:**
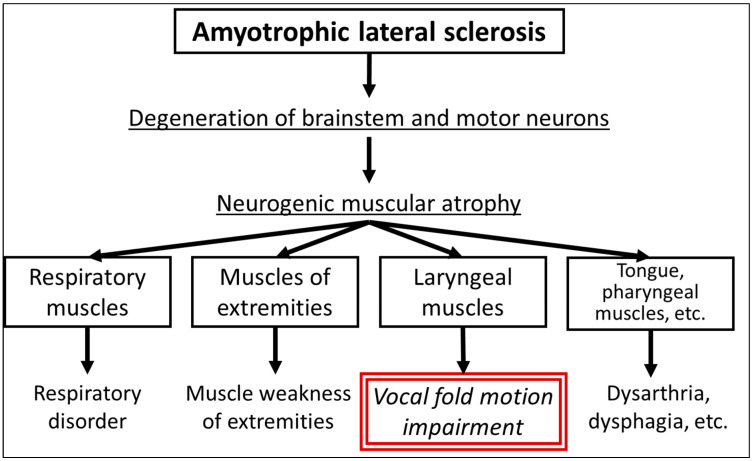
Pathophysiology of vocal fold motion impairment in amyotrophic lateral sclerosis.

ALS is a neurodegenerative disease that affects motor neurons and leads to progressive muscle weakness and atrophy. Patients with ALS can experience various vocal fold abnormalities, including paradoxical adduction patterns, aperiodic vocal fold vibrations, hyperadduction of the ventricular folds, and hypoadduction of the vocal folds. These abnormalities can lead to symptoms of dysphonia, dysarthria, and dysphagia, which are prevalent in the bulbar form of ALS that primarily affects the brainstem and cranial nerves [39,40]. The exact pathophysiology of vocal fold dysmotility in ALS is not fully understood; however, it is thought to have multiple causes involving both infranuclear and supranuclear origins, resulting in the degeneration of the motor neurons that control the muscles of the larynx [41,42,43,44] (Figure 2). 

Weakening of the upper airway muscles, including the vocal cord (fold) abductors, contributes to vocal fold movement impairment in ALS. VFMI in ALS can occur at any stage of the disease, and the symptoms range from hoarseness, hypophonia, and shortness of breath to acute dyspnea, laryngospasm, and stridor. Both unilateral and bilateral vocal cord (fold) dysfunction can manifest in ALS patients, contributing to a variety of laryngeal and respiratory symptoms. Involuntary vocal fold abduction dysfunction, including laryngospasm, can elicit dyspnea in ALS, with excessive saliva irritating the vocal folds, which is a commonly reported trigger [45]. 

Identification of these dysfunctions is important for the diagnosis and management of ALS, and comprehensive management strategies are essential to maintain quality of life, prevent complications, and improve patient outcomes [46]. When bilateral VFMI causes respiratory compromise in patients with ALS, tracheostomy to secure the airway should be considered, particularly when dysphagia is coexistent. However, because tracheostomy can impair patients’ swallowing function, continuous positive airway pressure or other measures are also an option, and shared decision making between the patients and medical professionals is performed to plan the best course of action.

### 3.2. Parkinson’s Disease (PD)

**Figure 3:**
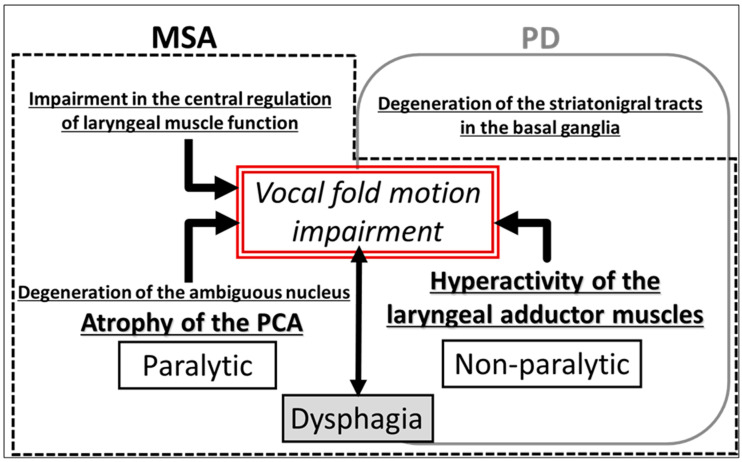
Pathophysiology of vocal fold motion impairment in Parkinson’s disease and multiple system atrophy. MSA, multiple system atrophy; PD, Parkinson’s disease; PCA, posterior cricoarytenoid muscle.

PD is characterized by neuromuscular impairments, including rigidity, tremor, and bradykinesia. Degeneration of the nucleus ambiguus, a region of the brainstem involved in motor control, may contribute to these symptoms. The pathogenesis of VFMI in PD is not completely understood, but it is thought to involve a combination of motor impairments, neuromuscular dysfunction, and degenerative changes in the brainstem nuclei responsible for laryngeal muscle control, which contribute to paralysis or weakness [47,48] (Figure 3).

In patients with PD, VFMI can manifest as hypophonia, monotone speech, vocal tremor, breathiness, and dysarthria [49]. In some cases, bilateral VFMI can occur. The prevalence of VFMI in patients with PD is relatively low compared with in that other neurodegenerative conditions [47]. However, bilateral VFMI, especially vocal fold abductor motion impairment, can be a life-threatening complication that may necessitate urgent interventions, such as tracheotomy or surgical treatments, to address airway obstruction [47,50]. Early recognition and appropriate management of VFMI in PD are important to improve voice quality, communication abilities, and overall quality of life for affected individuals.

### 3.3. Multiple System Atrophy (MSA)

MSA is a progressive neurodegenerative disorder characterized by autonomic dysfunction, parkinsonian disorder, and cerebellar dysfunction as the main symptoms. MSA is classified into parkinsonian (MSA-P) and cerebellar (MSA-C) variant types [9]. VFMI is a common issue in patients with MSA and is found in approximately half to two-thirds of the patients [9,51,52,53,54]. In particular, patients with MSA-P can develop VFMI earlier than patients with MSA-C because the spontaneous activity of intrinsic laryngeal muscles in patients with MSA-P can lead to an earlier onset of VFMI [9]. There are two plausible hypotheses for the pathophysiology of VFMI: (1) a severe loss of neurons in the nucleus ambiguus results in hypoactivity and neurogenic atrophy of the posterior cricoarytenoid muscle, which is the sole laryngeal abductor muscle, or (2) dystonia is induced by hyperactivity of the laryngeal adductor muscles [55,56] (Figure 3). 

Patients with MSA often have sleep-related respiratory disorders, including high-pitched inspiratory stridor and sleep apnea. Upper airway obstruction can be caused by bilateral VFMI. VFMI is exacerbated during sleep, and airway narrowing can worsen [7]. Sudden death is frequently reported in patients with MSA, and VFMI is one of the most common causes of this [57]. In addition, diazepam should not be administered to patients with VFMI because it can aggravate glottic closure in patients with MSA [58]. The severity of VFMI impacts the overall survival of MSA patients, with unilateral or bilateral VFMI being statistically associated with decreased survival rates [9,59]. Therefore, early detection through laryngological assessment is crucial for managing VFMI in patients with MSA.

### 3.4. Progressive Supranuclear Palsy (PSP)

PSP is characterized by the gradual worsening of symptoms over time due to damage to certain brain areas, affecting functions such as walking, thinking, swallowing, and eye movements [60]. The exact cause of PSP has not been completely clarified, but it involves aggregation of tau proteins found in the brain, leading to neuronal damage [61]. This condition is often misdiagnosed as PD initially, but it progresses more rapidly than PD. The mechanism behind VFMI in PSP involves neurodegenerative processes that affect the central nervous system [62]. In patients with PSP, the development of bilateral VFMI is linked to the progression of the disease, leading to respiratory issues like inspiratory stridor. This dysmotility is a result of the neurodegenerative changes in the brain, particularly affecting areas involved in motor control and coordination [63]. In some instances, emergency tracheostomy is necessary to alleviate the respiratory symptoms associated with vocal fold paralysis in patients with PSP [64]. This highlights the importance of monitoring and managing VFMI in patients with PSP to prevent serious respiratory complications.

### 3.5. Multiple Sclerosis (MS)

MS is a neurological condition that can affect the nerves that innervate the laryngeal muscles, leading to VFMI [65,66,67]. This vocal fold dysmotility can result in difficulties in speech, breathing, and swallowing. The impact of multiple sclerosis on vocal fold paralysis emphasizes the intricate relationship between NDDs and laryngeal function. 

## 4. Comparison of Patients with Vocal Fold Motion Impairment and PD or MSA

When comparing patients with VFMI and PD or MSA, VFMI is more frequently observed in MSA, and the irregular movement of the arytenoid cartilage may serve as a clinical marker to distinguish MSA from PD [8]. Airway narrowing due to VFMI is evidently more likely to occur in MSA [47]. In patients with MSA, the progression of VFMI and dysphagia was compared between patients with MSA-C and MSA-P. In MSA-C, worsening VFMI preceded the exacerbation of dysphagia, whereas, in MSA-P, severe dysphagia either preceded or occurred simultaneously with worsening VFMI [68]. Other differences in the clinical characteristics are summarized in Table 1. Compared with patients with PD, patients with MSA exhibited a longer duration from disease onset to VFMI, and their daily activities were more impaired at the onset of VFMI. At the onset of VFMI, dysphagia was notably severe in PD, while its severity varied among patients with MSA [47,68,69]. Inspiratory stridor was more prominent during the daytime in PD, while it predominated during sleep in MSA [47]. The effects of diazepam administration were entirely different between PD and MSA [58], and they also differed in their histological and laryngeal electromyographic findings [47,70].

## 5. Management and Treatment for Vocal Fold Motion Impairment

**Figure 4:**
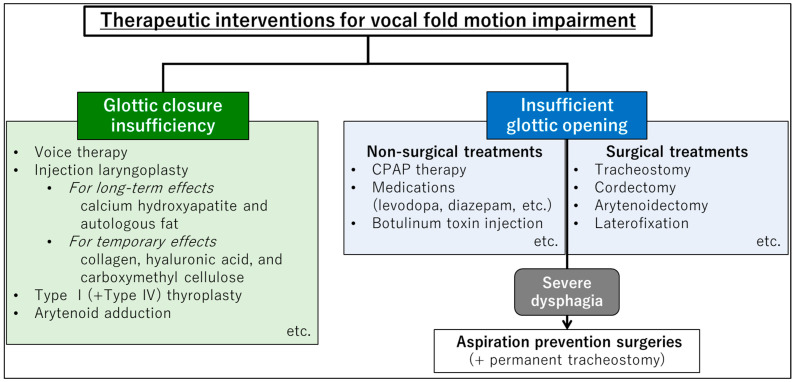
Therapeutic interventions targeting glottic closure insufficiency and insufficient glottic opening. CPAP, continuous positive airway pressure.

VFMI manifests with challenges in both glottal closure and opening, demanding distinct therapeutic strategies for each. Hereafter, we detail the therapeutic interventions targeting glottic closure insufficiency and insufficient glottic opening (Figure 4).

### 5.1. Treatments for Glottic Insufficiency

Treatment options for glottic insufficiency range from surgical interventions, including injection laryngoplasty and medialization thyroplasty, to behavioral therapies, such as voice therapy. These treatments aim to improve glottic closure to prevent aspiration and enhance vocal function. 

Speech therapy, particularly techniques such as Lee Silverman Voice Treatment, can help improve vocal projection, intonation, and overall voice quality in patients with PD. This therapy focuses on increasing vocal loudness and clarity, enhancing communication skills, and addressing voice-related issues through one-on-one training sessions [78].

Injection laryngoplasty (augmentation laryngoplasty) is often helpful for hypoactive neurolaryngological disorders that result in glottal insufficiency and a weak voice. Injection laryngoplasty can be performed with a temporary injectable in an office setting that can address glottal closure, reduce glottal atrophy, and improve voice strength. Injectable materials are currently categorized as either temporary or long-term. Options for temporary materials include those derived from collagen, hyaluronic acid, and carboxymethyl cellulose, whereas calcium hydroxyapatite and autologous fat are available for long-term use. If temporary injection improves symptoms, longer term materials and more permanent interventions should be considered [79,80]. In cases of glottic insufficiency caused by unilateral vocal fold paralysis, surgical treatments using external approaches, such as type I (+IV) thyroplasty and arytenoid adduction, as well as injection laryngoplasty, are viable options [81]. Injection laryngoplasty is a minimally invasive procedure involving injections into the vocal folds under local anesthesia, whereas external approach surgeries are more invasive and permanent, suitable for cases wherein irreversible causes of severe glottal incompetence need to be addressed surgically [82].

### 5.2. Treatments for Insufficient Glottic Opening

Insufficient glottic opening refers to glottic narrowing due to vocal fold movement disorders such as bilateral vocal fold paralysis and bilateral abduction disorder. To treat insufficient glottic opening due to VFMI, various options are available based on the severity and underlying cause of the condition. Treatment approaches for managing VFMI in patients with NDDs include continuous positive airway pressure (CPAP) therapy, botulinum toxin (BTX) injection, tracheostomy, vocal fold surgery, or a combination of interventions can be considered [83,84]. If noninvasive interventions, such as drug therapy and CPAP, do not alleviate airway narrowing, surgical treatments should be performed.

CPAP has been shown to effectively manage glottic narrowing without surgical intervention in patients with bilateral vocal fold paralysis, particularly in patients with NDDs [83]. It involves wearing a mask over the nose and/or mouth, which delivers a continuous flow of air to keep the airway open and to prevent obstruction. CPAP reduces intratracheal negative pressure, thereby decreasing the transglottic pressure gradient through glottic stenosis during inspiration, reducing the likelihood of laryngeal adductor activation [8,85,86]. BTX injections can be used to temporarily weaken the function of the overactive vocal folds in cases of spasmodic dysphonia or adductor-type vocal fold paralysis. Although its effects diminish over the course of several months, BTX can improve airway narrowing and reduce the symptoms of dyspnea [87,88]. When glottic narrowing occurs in patients with PD due to VFMI, adjusting the dosage of levodopa may improve the symptoms [89].

Surgical treatments for insufficient glottic opening include various options aimed at improving airway patency. It should be noted that treatments to widen the glottis and secure the airway, other than tracheotomy, tend to worsen the quality of the voice because of glottic insufficiency after surgeries, and make the patient more susceptible to aspiration because the glottis does not close during swallowing.

- Tracheostomy is considered an essential intervention in managing bilateral VFMI, especially in situations where there is a risk of airway compromise due to the inability of the vocal folds to open properly during breathing. This surgical opening bypasses the narrowed portion of the airway, allowing for adequate breathing and preventing life-threatening obstruction of the airway [84,90].
- Cordectomy is an irreversible method of widening the glottis, involving excision of a part of the vocal fold, vocal ligament, or thyroarytenoid muscle. This improves airway patency and alleviates respiratory symptoms [91,92].
- Arytenoidectomy is a surgical procedure in which the arytenoid cartilage, one of the cartilages in the larynx, is either completely or partially removed. This procedure is typically performed to address certain conditions affecting the larynx, such as insufficient glottic opening. However, this is not commonly performed on bilateral vocal folds because of the potential risks and impact on voice and swallowing functions [93,94].
- Laterofixation is a reversible method for the treatment of bilateral vocal fold paralysis in both adults and children. This procedure may be applied independently or in combination with other laryngeal microsurgery methods, unilaterally or bilaterally. Suture laterofixation is highlighted as an important option in the treatment of bilateral VFMI, and it avoids the long-term consequences of ablative procedures like arytenoidectomy [91,95,96].

### 5.3. Treatment for Insufficient Glottic Opening with Severe Dysphagia

When severe dysphagia is present, in addition to insufficient glottic opening, it is necessary to provide a means of nutrition (tube feeding, gastrostomy, etc.) as well as to secure the airway. Since tracheostomy worsens swallowing function, aspiration prevention surgery can be considered in patients with insufficient glottic opening and severe dysphagia. Although patients who undergo aspiration prevention surgeries eventually lose their vocal function, they are able to practice oral nutrition or oral intake by creating a structure preventing aspiration, which improves the patients’ quality of life [90,97,98,99,100].

## 6. Discussion

To inform healthcare professionals about VFMI in patients with NDDs, we have compiled this review article. Many physicians may have limited experience with NDDs and may be largely unaware of VFMI in these patients. Consequently, when examining patients who visit their clinic due to voice or respiratory problems, even if VFMI were found, physicians may not consider it as being related to NDDs. This review paper provides a comprehensive explanation of the pathophysiology and management of VFMI due to NDDs, offering advantages over previous literatures. Additionally, as clinically significant knowledge, it delineates differences in the management of VFMI between PD and MSA, which present similar findings. 

When the vocal folds have trouble moving smoothly, voice, swallowing, and breathing are impaired, which not only reduces the patients’ quality of life, but can also be a critical and life-threatening issue. Patients with NDDs often experience progressive worsening of various symptoms over time and, aside from breakthrough molecular-targeted drugs, there are currently no viable drugs or therapies for alleviating these symptoms. Therefore, when VFMI is identified in a patient, it is essential to consider the progression of the disease and discuss appropriate measures to address it.

One must be aware that the management of bilateral severe VFMI in patients exhibiting parkinsonian symptoms is not the same for patients with PD and patients with MSA. In PD, airway narrowing is influenced by hyperactivity of the laryngeal adductor muscles and can be improved by the administration of diazepam, a drug that alleviates hypertonia. In contrast, in MSA, diazepam administration may worsen VFMI, resulting in the need for emergency measures for airway narrowing such as endotracheal tube insertion or tracheostomy.

In cases where VFMI (bilateral vocal fold abductor paralysis) induces airway narrowing, CPAP or tracheostomy may be necessary; nevertheless, neither CPAP nor tracheostomy can offer a permanent solution. Even when CPAP is used, the mask must be removed during meals, which may exacerbate respiratory disturbances during that time. Even if the airway is secured by tracheostomy, swallowing function may deteriorate with the placement of a tracheostomy tube. Consequently, if swallowing function is impaired, a tracheostomy tube for speaking cannot be utilized. In evaluating patients presenting with VFMI, it is important to account for the potential presence of underlying NDDs. Moreover, when addressing airway narrowing due to VFMI in patients with NDDs, it is appropriate to consider a broad perspective that encompasses not only current problem-solving but also future implications for vocal and swallowing functions, as well as the quality of life of patients. It is important to carefully consider the preferences of patients and their families when determining the appropriate course of action. Future studies are needed to evaluate the progression following treatment for VFMI in patients with NDDs. As for limitations, the details of the diagnostic and surgical procedures for VFMI were not included in this review article, and we refer the reader to more specialized books and articles for this information.

## 7. Conclusions

This review outlined the clinical examination approaches and management strategies for VFMI in patients with NDDs from the perspective of an otolaryngologist. To address VFMI in patients with NDDs, it is desirable to consider a broad perspective that encompasses future implications for vocal, respiratory, and swallowing functions, as well as patients’ quality of life. It is important to carefully consider the preferences of patients and their families while deliberating on the appropriate course of action.

## Figures and Tables

**Table 1:** Comparison of clinical findings in patients with Parkinson’s disease and multiple system atrophy who developed vocal fold motion impairment. PD, Parkinson’s disease; MSA, multiple system atrophy; VFMI, vocal fold motion impairment; TA, thyroarytenoid muscle; LCA, lateral cricoarytenoid muscle; PCA, posterior cricoarytenoid muscle.

	PD	MSA
Age (year) [47,68,69]	68.7 ± 9.2	62.8 ± 8.0
Sex [47,68,69]	Male > Female	Male > Female
Onset to VFMI (year) [47,68,69]	10.4 ± 5.6	7.6 ± 3.2
Hoehn and Yahr scale [47,68] (Usually applied to PD)	Stage 5: 100%	Stage 3: 6.3% Stage 4: 43.7% Stage 5: 50%
Findings of VFMI [8,71,72,73,74]	Vocal fold abductor paralysis Glottic insufficiency	Vocal fold abductor motion impairment Paradoxical vocal fold movement
Degree of dysphagia at detection of VFMI [68]	Severe dysphagia in most cases	Varying degrees from normal to severe
Inspiratory stridor [47]	Daytime > Bedtime	Daytime < Bedtime
Diazepam test [58]	Improvement or no change in VFMI	VFMI worsening
Histology of the PCA [47,70]	No morphological abnormalities	Neurogenic muscular atrophy
Electromyography [75,76,77]	Hyperactivity of the laryngeal adductor muscles	Dystonic activation of TA and LCA Paradoxical activation of TA Reduced activity of PCA during sleep

## Data Availability

The datasets used and analyzed during the current study are available from the corresponding author upon reasonable request.
